# Outcomes of Primary Mucosal Head and Neck Squamous Cell Carcinoma in Solid Organ Transplant Recipients

**DOI:** 10.7759/cureus.24305

**Published:** 2022-04-20

**Authors:** Marissa Gilbert, Evan Liang, Pin Li, Reena Salgia, Marwan Abouljoud, Farzan Siddiqui

**Affiliations:** 1 Radiation Oncology, Henry Ford Health, Detroit, USA; 2 Public Health Sciences, Henry Ford Health, Detroit, USA; 3 Gastroenterology and Hepatology, Henry Ford Health, Detroit, USA; 4 Transplant and Hepatobiliary Surgery, Henry Ford Health, Detroit, USA

**Keywords:** locoregional recurrence, radiation therapy, organ transplant, head and neck, squamous cell carcinoma

## Abstract

Introduction

Patients who undergo solid organ transplants have a higher risk of developing malignancies and subsequent recurrences. Clinical outcomes in transplant recipients with primary mucosal head and neck squamous cell carcinoma (HNSCC) are not well described in the published literature. Therefore, we retrospectively studied the outcomes in this group of patients.

Methods

This Institutional Review Board (IRB)-approved analysis included patients who had previously undergone solid organ transplants and subsequently were diagnosed with primary mucosal HNSCC between 2006 and 2021. Our institutional database of solid organ transplant recipients was cross-referenced with our head and neck cancer database to identify the patients included in this cohort. In addition, Kaplan-Meier analyses were performed to calculate overall and disease-free survival.

Results

Of 1,221 patients, 20 met the inclusion criteria. The median time from organ transplant to HNSCC diagnosis was 5.9 years (range: 0.5-18.5 years). A total of 11 (55.0%) and 9 (45.0%) patients presented with localized and locally advanced disease, respectively. Two-year overall and disease-free survivals were 59.1% and 73.5%, respectively. After initial treatment, six (30.0%) patients experienced a recurrence. All patients who developed a recurrence died within the follow-up period. The median time of death after recurrence for all six patients was 11.5 months (range: 2-22 months).

Conclusion

This series highlights a high mortality rate following recurrence among patients with primary mucosal HNSCC and a solid organ transplant history. A better understanding of how solid organ transplant history adversely impacts the course of HNSCC could help properly guide treatment, follow-up, and survivorship decisions.

## Introduction

Individuals with a history of solid organ transplants are noted to have an increased incidence of cancer diagnoses [1]. Cutaneous squamous cell carcinomas (SCC) are reported to occur up to 65 times more commonly in these patients compared to the general population [2-5]. In cutaneous SCC with primary tumors located in the head and neck, immunosuppressive therapy has been associated not just with increased incidence but also with increased recurrence and poorer survival [6]. Aerodigestive mucosal head and neck squamous cell carcinoma (HNSCC) incidence has also been shown to be associated with a history of organ transplants, particularly oral cavity and oropharyngeal cancers [7]. Relative to cutaneous SCCs of the head and neck, outcomes among patients with mucosal HNSCC and a history of solid organ transplant are less well-studied. Our aim was to retrospectively characterize this patient cohort treated at an urban transplant center with a dedicated head and neck oncology team to uncover possible trends that could help guide treatment decisions for these challenging patients in a multidisciplinary setting.

## Materials and methods

This retrospective analysis was performed after obtaining approval from the IRB (approval number 8751). Initially, patients were identified in our institutional organ transplant database. Next, the Epic (Epic; Madison, WI) electronic medical records system's SlicerDicer function was used to identify patients with mucosal HNSCC. The identified patient records were then cross-referenced with our head and neck cancer database (REDCap 10.6; Nashville, TN). Patients 18 years and older diagnosed with primary HNSCC over a 15-year period between March 2006 and March 2021 were included. The analysis included patients who had previously undergone a solid organ transplant and were subsequently diagnosed with a primary mucosal HNSCC. Patients with bone marrow transplants, primary cutaneous malignancies, non-SCC histologies, and external lip primary tumors were excluded from the analysis.

Patient characteristics, including immunosuppression regimens, tumor staging, treatment details, and cancer control outcomes, were queried from the database. The staging was performed according to the previous American Joint Committee of Cancer (AJCC) 7th edition, with special regard to human papillomavirus positive oropharyngeal cancer staging, as neck nodal and overall staging have significant differences in the current AJCC 8th edition. Overall survival (OS) was defined as the date of diagnosis to death censored at the last follow-up. Locoregional control (LRC) was defined as the date of diagnosis to local or regional failure censored at death or last follow-up. OS and LRC were estimated using the logrank test to compare different cohorts using Kaplan-Meier methods. In addition, the time-dependent Cox proportional hazard model was used to assess the impact of LRC on OS. All analysis was done in R 4.0.4, and statistical tests were two-sided with an α (significance) level of 0.05.

## Results

Patient characteristics

Out of 1,221 patients in the head and neck cancer database, 23 patients met inclusion criteria. Three patients were excluded due to a lack of treatment and/or follow-up information, resulting in a cohort of 20 patients. The clinical features of the patients are shown in Table 1. The median age of diagnosis was 59.5 years (range: 47-74 years), and most patients were male (n=16, 80.0%). Of these patients, 12 (60.0%) had undergone a liver transplant, four (20.0%) had a kidney transplant, and one (5.00%) had a lung transplant. Three patients (15.0%) had received multiple transplants, with two patients having received both a liver and kidney transplant and one patient receiving a kidney and heart transplant. History of tobacco use was common among this cohort, with 14 (70.0%) patients having >10 pack-year smoking history. Alcohol consumption was also common, with 12 (60.0%) patients having regular usage documented in their medical records. 

**Table 1:** Patient characteristics. HNSCC: Head and neck squamous cell carcinoma.

		Number of patients (n=20)	%
Age	<50	1	5.00
	>=50 and <60	9	45.0
	>=60 and <70	8	40.0
	>=70	2	10.0
Gender	Male	16	80.0
	Female	4	20.0
Smoking history	<10 pack years	6	30.0
	>=10 pack years	14	70.0
Alcohol history	No	8	40.0
	Yes	12	60.0
Type of transplant	Liver	12	60.0
	Kidney	4	20.0
	Lung	1	5.00
	Liver and kidney	2	10.0
	Kidney and heart	1	5.00
Time from transplant to HNSCC diagnosis	<24 months	3	15.0
	>=24 and <48 months	3	15.0
	>=48 and <72 months	2	10.0
	>=72 and <96 months	6	30.0
	>=96 and <120 months	0	0.00
	>=120 months	6	30.0
Immunosuppression prior to HNSCC diagnosis	Calcineurin inhibitor	15	75.0
	mTOR inhibitor	2	10.0
	Both	3	15.0
Immunosuppression after HNSCC diagnosis	No change in use	11	55.0
	Change in use	9	45.0
Primary tumor site	Oropharyngeal	7	35.0
	Oral cavity	7	35.0
	Laryngeal	5	25.0
	Hypopharyngeal	1	5.00
Grade	Stage 0	1	5.00
	Stage I	7	35.0
	Stage II	3	15.0
	Stage III	3	15.0
	Stage IV	6	30.0
Treatment modality	Surgery alone	7	35.0
	Post-operative radiation/chemoradiation	5	25.0
	Definitive chemoradiation	5	25.0
	Definitive radiation	3	15.0

For the immunosuppression regimen prior to HNSCC diagnosis, 15 (75%) were on calcineurin inhibitors, two (10%) were on mTOR inhibitors, and three (15%) were on both. The median time to HNSCC diagnosis after receiving a solid organ transplant was 5.9 years (range: 0.5-18.5 years). Of the 15 patients previously on calcineurin inhibitors, nine patients continued their regimens without change, one had lowered dosing, three switched to mTOR inhibitor, one remained on mycophenolate mofetil alone, and one came off immunosuppression entirely. The two patients on mTOR inhibitor alone continued their regimens; the three patients taking both calcineurin inhibitors and mTOR inhibitors all discontinued their calcineurin inhibitors and continued with mTOR inhibitor alone.

Disease characteristics

The primary tumor sites were predominantly oropharyngeal (n=7, 35.0%), oral cavity (n=7, 35.0%), laryngeal (n=5, 25.0%), and hypopharyngeal (n=1, 5.00%) cancers. The cohort included 11 patients (55.0%) who presented with localized disease (stages 0, I, II), and the remaining nine patients (45.0%) had presented with locally advanced disease (stages III, IVa-b). No patients had distant metastases at the time of diagnosis. 

Treatment details 

Twelve patients (60.0%) had primary surgical resection with or without neck lymph node dissection. Of these, five (41.7%) received adjuvant radiation with or without concurrent chemotherapy, while the remaining seven patients (58.3%) received no adjuvant treatment. Patients treated with adjuvant radiation were prescribed a median dose of 60Gy (range: 54-66Gy). Nine patients were treated with primary radiation with or without concurrent chemotherapy and were prescribed a median dose of 70Gy (range: 40-70Gy).

Outcome 

**Figure 1:**
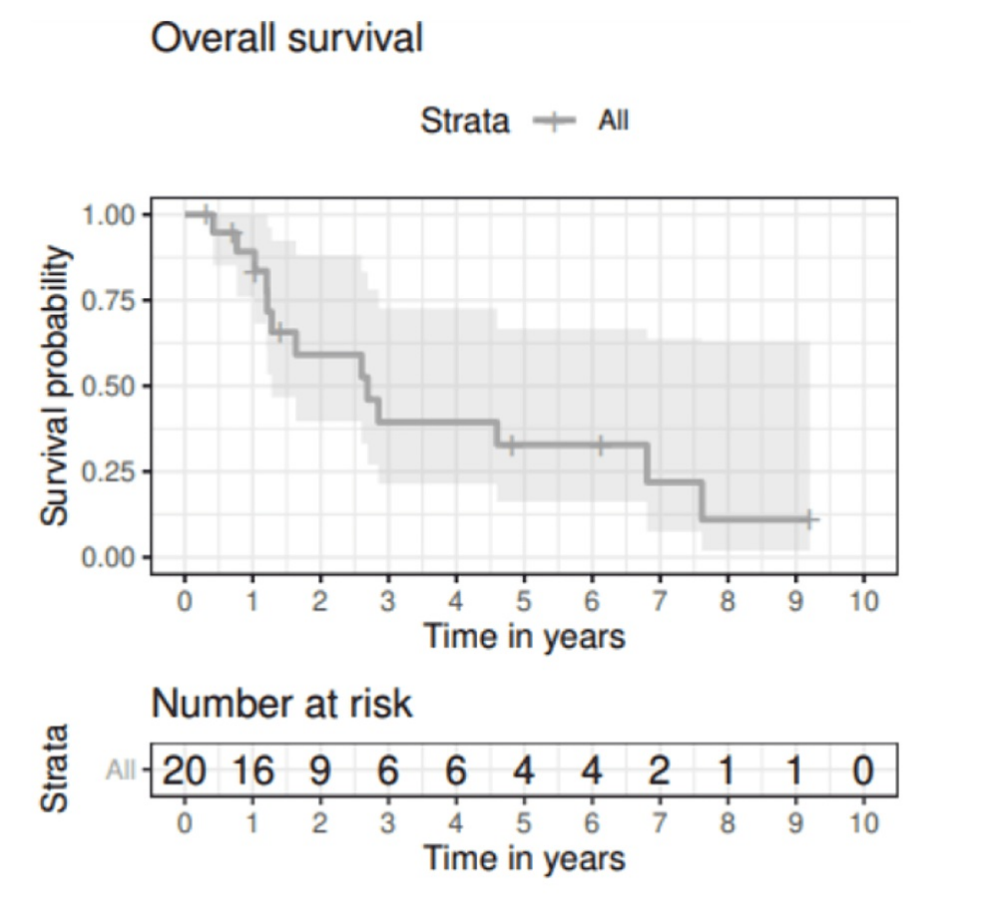
Overall survival for solid organ transplant patients subsequently diagnosed with HNSCC. HNSCC: Head and neck squamous cell carcinoma.

At a median follow-up of 6.1 years (range: 0.3-9.2 years), the median OS for the cohort was 2.7 years (95% CI: 1.28-NA). Figure 1 presents OS following completion of primary treatment. This corresponded to two-year and five-year OS rates of 59.1% (95% CI: 39.7-88.1) and 32.8% (95% CI: 16.2-66.6), respectively. The two-year survival rate was 76.2% (95% CI: 52.1-100.0) for localized disease versus 38.1% (95% CI: 15.7-92.4) for locally advanced disease. At five years, these survival rates were 38.1% (95% CI: 15.7-92.4) and 25.4% (95% CI: 07.7-83.8). Differences in survival rates between groups did not reach statistical significance by the logrank test (p = 0.31). 

**Figure 2:**
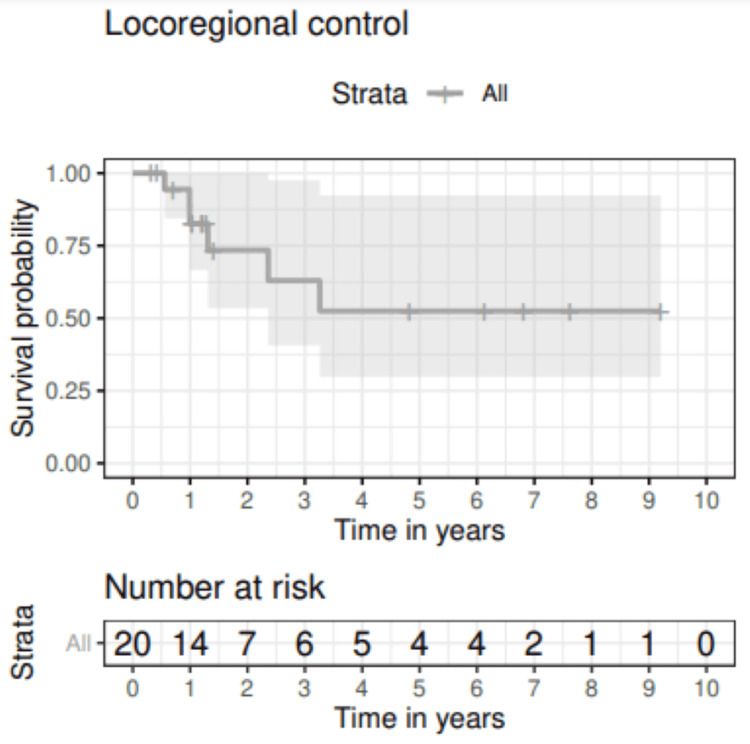
Locoregional control for solid organ transplant patients diagnosed with HNSCC that later presented with recurrence. HNSCC: Head and neck squamous cell carcinoma.

Figure 2 shows locoregional control of the entire cohort; 73.5% (95% CI: 53.5-100) and 52.5% (95% CI: 29.8-92.4) of patients were disease-free at two and five years, respectively. After initial treatment, six patients (30.0%) experienced a locoregional recurrence in the head and/or neck at a median of 13.5 months (range: 6-39 months). Characteristics of these patients are shown in Table 2. Notably, distant failure occurred in two patients after locoregional recurrence. No other instances of distant failure were observed. Salvage or palliative treatment was initiated for five of six patients who experienced recurrence, and four completed their planned treatment courses. All patients who experienced locoregional recurrence died within the follow-up period. The median time to death after recurrence was 11.5 months (range: 2-22 months). 

**Table 2:** Recurrent patient characteristics. ND: Neck dissection; RT: Radiation therapy; WLE: Wide local excision.

Patient	Type of Transplant	Age, Sex	Tumor Site	Stage	Treatment Modality	Type of Immunosuppression, Change in Immunosuppression after HNC Diagnosis	Recurrence	Recurrence Treatment Modality	Initial Diagnosis to Recurrence (months)	Subsequent Distant Failure	Recurrence to Death (months)
1	Liver	61, M	Oral Cavity	III	Chemo + RT (40Gy/20fx, concurrent carboplatin)	Calcineurin, Stopped Immunosuppression	Primary + Neck	Palliative RT (37.5Gy/15fx – elected for hospice after 6fx)	28	No	2
2	Kidney	57, M	Larynx	I	Definitive RT (63GY/28fx)	Calcineurin, Stopped Calcineurin and Started mTOR	Neck	Palliative Chemo (cetuximab/ carboplatin/5-FU)	39	Yes	16
3	Liver	58, M	Oropharynx	II	Definitive RT (67.84GY/32fx)	Calcineurin and mTOR, Stopped Calcineurin and Continued mTOR	Primary	Surgery Alone (Biopsy Only)	11	No	22
4	Liver	53, F	Oral Cavity	I	Surgery Alone (WLE + ipsilateral ND)	Calcineurin, Stopped Immunosuppression	Primary + Neck	Surgery Alone (Repeat ND)	12	No	7
5	Liver	63, M	Oropharynx	IVa	Chemo + RT (70Gy/35fx, concurrent cetuximab)	Calcineurin, No Change	Neck	Palliative RT (20Gy/5fx)	6	No	3
6	Kidney	68, M	Larynx	I	Surgery Alone (WLE)	Calcineurin, No Change	Primary	Definitive RT (63Gy/28fx)	15	Yes	16

## Discussion

Solid organ transplant history and associated long-term immunosuppressive therapy have been implicated in both the increased incidence and aggressive course of cutaneous SCCs [2-6]. Mechanisms proposed for this increased susceptibility include interactions with ultraviolet radiation-induced carcinogenesis or oncogenic viruses, the latter being also implicated in mucosal HNSCC [5]. In this study, we observed a two-year OS of 38.1% in patients with locally advanced mucosal HNSCC. This is lower than the three-year OS of 65% and two-year OS of 63% noted in the European Organisation for Research and Treatment of Cancer (EORTC) 22931 and Radiation Therapy Oncology Group (RTOG) 9501 studies of non-transplant HNSCC patients receiving trimodality therapy, as well as the two-year OS of 54% in the meta-analysis of chemotherapy in head and neck cancer (MACH-NC) of patients receiving definitive chemoradiation [8-14]. This difference might be explained by the high mortality among patients who had progression of disease in our study. 

This cohort had a locoregional recurrence rate of 30.0%, which is within the expected range of 21.1%-35.8% in previously published randomized studies in non-transplant HNSCC [11,12]. Among our patients who had a locoregional recurrence, none were alive at the last follow-up. This contrasts with a reported OS of 39% in a prospective randomized trial of HNSCC patients who received salvage surgery alone for recurrence. However, this may have created a selection bias of those patients who were healthy enough for salvage surgery [13]. In another multi-institutional study focusing on head and neck re-irradiation, median survival was 16.5 months, and the actuarial survival rate was 40.0% at two years [14].

Our findings are comparable to the limited institutional experiences that have been published on mucosal HNSCC following solid organ transplant. When evaluating experiences limited to primary mucosal HNSCC only, specifically oropharynx, hypopharynx, oral cavity, and larynx, sample sizes ranged from nine to 33 [15-19]. Information regarding comparable studies is summarized in Table 3. Our interval to HNSCC diagnosis was 5.9 years, which is consistent with previous case series reporting values ranging from 2.0 to 9.4 years. In addition, we noted a median OS of 2.7 years, which is within the range of 1.8-3.8 years found in previously published reports. Our five-year OS rate of 32.8% similarly falls within the published range of 11%-42%. One finding of note in our study is the poor survival rate in patients who experienced a recurrence, regardless of tumor site and initial stage. Patients who experienced local recurrences were significantly more likely to die, with none of these patients being alive at the end of the follow-up period. This is despite the fact that local treatment was offered to all patients. Some of these salvage treatments can be morbid, whether it is re-irradiation, chemotherapy, immunotherapy, or surgery in a previously treated area [20-22].

**Table 3:** Comparison of previous studies involving head and neck squamous cell carcinoma after solid organ transplant.

Study	N	HNSCC	Five-year Survival	Comments
Alsidawi S et al. (2017) [15]	33	30	39%	Included 2 SCC salivary gland malignancies and 1 unknown primary
Scheifele C et al. (2005) [16]	13	13	42%	Included only liver transplants
Coordes A et al. (2016) [17]	33	33	34%	Included only liver transplants
Öhman J et al. (2015) [18]	Oral Cavity = 17, Lip = 34	12	Oral Cavity = 27%, Lip = 61%	Included a comparison between 17 oral cavity malignancies and 34 lip malignancies. Oral cavity included 5 non-SCC salivary gland malignancies. Excluded patients with multiple transplants.
Lin NC et al. (2019) [19]	9	8	11%	Included only liver transplants and 1 patient with a salivary gland malignancy
Gilbert M et al. (2021)	20	20	33%	Current Study

The particular classes of drugs used for immunosuppression in our cohort are also worth examining. Calcineurin inhibitor-based regimens have been associated with higher rates of SCCs [5,23,24]. Randomized clinical trials and meta-analyses in kidney transplant patients have shown that switching from a calcineurin-based regimen could increase the cutaneous SCC-free survival [25,26]. In addition, mTOR inhibitors, independent of the organ transplant setting, have also been considered cytostatic therapeutics for HNSCC [27]. One meta-analysis looked at trials examining the use of mTOR inhibitors as monotherapies, in conjunction with chemoradiation, and in conjunction with EGFR inhibitors; it was concluded that survival was highest when mTOR inhibitors were combined with chemoradiation [28]. This would make mTOR inhibitors a potentially attractive alternative to calcineurin-based immunosuppression in patients subsequently diagnosed with HNSCC. In our cohort, nine patients had adjustments to their immunosuppressive regimens; of these, three patients were switched from calcineurin inhibitor to mTOR inhibitor. The sample sizes here were too small to conclude the effects of these changes on recurrences, but adjustment of immunosuppression should be a focus in any future analyses. 

Also of relevance to our cohort, checkpoint inhibition immunotherapy may be more complex in patients on immunosuppression for an organ transplant [29]. This is especially important given the increasing role of immunotherapy in HNSCC patients as a preferred first-line systemic therapy option for patients with recurrent or metastatic disease. The KEYNOTE-048 trial demonstrated superior survival with pembrolizumab in combination with chemotherapy (platinum and 5-fluorouracil) compared to chemotherapy alone or chemotherapy plus cetuximab [30]. The trial also showed the efficacy of single-agent pembrolizumab in patients with tumors staining positive for programmed death-ligand 1. Regardless of the approach, the patient selection remains crucial in managing recurrent HNSCC. The high mortality seen in our cohort suggests that organ transplant and long-term immunosuppression should be taken into account when deciding on appropriate local or systemic therapies for recurrences.

Our institutional experience of treating patients with HNSCC who had a previous solid organ transplant is a limited descriptive review of a select group of patients due to the rarity of this diagnosis. In addition, this specific cohort includes patients with various primary tumor sites and wide distribution of cancer stages. A larger patient sample would allow matching with primary mucosal HNSCC patients who do not have a transplant history and could refine the comparison of clinical outcomes.

## Conclusions

This single-institution retrospective study reviewed outcomes among patients who developed primary mucosal HNSCC following a solid organ transplant. Overall survival was poor and may have been driven largely due to low survival following recurrence. These outcomes are consistent with previously published analyses and, together, highlight the need for matched cohorts for better comparison. Such data can then be used to properly inform multidisciplinary treatment discussions.
